# The Chem-Sex Inventory Scale (CSI): A Tool to Assess the Mental Health Risk of Chemsex Behaviors in Men Who Have Sex with Men

**DOI:** 10.3390/nursrep14030166

**Published:** 2024-09-04

**Authors:** Pablo Del Pozo-Herce, Enrique Baca-García, Antonio Martínez-Sabater, Rubén Pérez-Elvira, Vicente Gea-Caballero, Elena Chover-Sierra, Pedro José Satústegui-Dordá, Alberto Tovar-Reinoso, Francisco José Rodríguez-Velasco, Mercedes Sánchez-Barba, Jesús Pérez, Raúl Juárez-Vela

**Affiliations:** 1 Doctorate Program in Psychology, Autonomous University of Madrid, 28049 Madrid, Spain; pablo.pozo@quironsalud.es; 2 Department of Psychiatry, Fundación Jiménez Díaz University Hospital, 28040 Madrid, Spain; ebaca@quironsalud.es; 3 Instituto de Investigación Sanitaria de la Fundación Jiménez Díaz, 28040 Madrid, Spain; 4 UNIE Universidad, 28040 Madrid, Spain; alberto.tovar@universidadunie.com; 5 Nursing Care and Education Research Group (GRIECE), GIUV2019-456, Nursing Department, Universitat de Valencia, 46010 Valencia, Spain; elena.chover@uv.es; 6 Care Research Group (INCLIVA), Hospital Clínico Universitario de Valencia, 46014 Valencia, Spain; 7 Faculty of Psychology, Potificial University of Salamanca, 37007 Salamanca, Spain; rperezel@upsa.es; 8 Research Group Community Health and Care, Faculty of Health Sciences, International University of Valencia, 46014 Valencia, Spain; vagea@universidadviu.com; 9 Internal Medicine, Consorci Hospital General Universitari de Valencia, 46014 Valencia, Spain; 10 SAPIENF (B53_23R) Research Group, Faculty of Health Sciences, University of Zaragoza, 50009 Zaragoza, Spain; pjsd@unizar.es; 11 Biopsychosocial Research Group (GIBIPSO), Faculty of Medicine and Health Sciences, University of Extremadura, 10003 Caceres, Spain; fcorodriguezv@unex.es; 12 Department of Statistics, Faculty of Medicine, University of Salamanca, 37007 Salamanca, Spain; 13 Prevention and Early Intervention in Mental Health (PRINT), Faculty of Medicine, Biomedical Institute of Salamanca, University of Salamanca, 37007 Salamanca, Spain; jesusperez@usal.es (J.P.); raul.juarez@unirioja.es (R.J.-V.); 14 Department of Medicine, Faculty of Medicine, University of Salamanca, 37007 Salamanca, Spain; 15 Research Group in Care, Faculty of Health Sciences, University of La Rioja, 26004 Logroño, Spain

**Keywords:** illicit drugs, mental health, psychiatry, substance-related disorders

## Abstract

**Background**: Chemsex has been defined as the deliberate use of drugs for prolonged sexual intercourse between gay and bisexual men and other men who have sex with men (MSM). Drugs associated with chemsex can trigger mental health problems such as anxiety, depression, risk of psychosis and suicidal ideation, social isolation, stigmatization, and even loss of impulse control and lack of coping strategies. Currently, the increase in illicit drugs in a sexual context is considered an outbreak of a public health emergency. **Objective**: The aim of this study is the construction and validation of the Chem-Sex Inventory (CSI), a new scale to assess the mental health risk of chemsex behaviors. **Methods**: A cross-sectional design was conducted to study 563 participants. Data were collected through an online questionnaire between January and April 2023, and the construct validity of the CSI was assessed through exploratory and confirmatory factor analysis. **Results**: The sample was, on average, 36 years old (SD: ±9.2). The majority of gender identity was cisgender (97.7%). A factor structure was found that can be summarized in four dimensions: emotional instability, risk of psychosis, altered body perception, and risk of suicide. The confirmatory factor analysis (CFA) presents adequate reliability values, with a Cronbach’s alpha above 0.87 for all dimensions and a McDonald’s omega above 0.88 with a good fit of the 42 items. **Conclusions**: Our study has shown that the Chem-Sex Inventory (CSI) scale has factorial validity and could be used in clinical practice and research to measure the behavioral contribution of the chemsex phenomenon in MSM.

## 1. Introduction

The assessment of sexualized substance use during sexual activity has gained increasing attention in recent years. This phenomenon, known as chemsex, derives from the words “chems” (chemical substances) and “sex” [1]. Chemsex is defined as “the deliberate use of drugs to prolong sexual intercourse among gay and bisexual men and other men who have sex with men (MSM)” [1,2,3]. The longer the duration of chemsex, the greater the exposure to mental health risks, harms associated with substance use [4], and sexually transmitted infections (STIs). The European ChemSex Forum further describes chemsex as “a particular type of sexualized substance use practice among gay and bisexual men, trans and non-binary individuals, and MSM who engage in casual or uncommitted sexual encounters” [2,5].

Since 2010, there has been an observed increase in recreational drug use in sexual contexts among gay men, bisexuals, and MSM in Spain, particularly in major cities like Madrid and Barcelona [3,4]. Alarmingly, there has been a 702% increase in the number of people treated for chemsex addiction in the Community of Madrid, from 50 in 2017–2018 to 351 between January 2021 and July 2022 [6]. This trend has emerged as a public health problem closely linked to risky sexual practices [7]. Consequently, there has been a notable rise in the demand for emergency and mental health services due to substance use associated with chemsex [8].

The term is associated with substance use, especially methamphetamine, mephedrone, and GHB/GBL [1,9], depending on the context and geographic location [10]. It can last several hours or even days, with time being a key factor. The effects of the consumption of several substances at the same time can be potentiated and prolonged. In addition, access to drugs in illicit markets can increase the possibility of accessing impure drugs or drugs other than those they choose to consume, which can lead to poly-drug use, increasing the risk of acute side effects and increasing the magnitude of the problem. This can negatively affect emotional management and impulse control [11], contributing to the development of dependence on substances associated with chemsex [12] and impairing mental health, as well as generating public health, sexual, occupational, and social problems [13,14].

A strong association has been seen between substance use in the chemsex context and sexual behavior [3,15,16,17]. Drugs associated with chemsex can also trigger mental health problems such as anxiety, depression [18], risk of psychosis and suicidal ideation, social isolation, stigmatization, and even loss of impulse control and lack of coping strategies [19]. However, this substance use in a sexual context is new, has attracted a great deal of interest, and is currently considered a public health emergency [8]. The patterns of consumption and sexual behavior in people who practice chemsex include prolonged sexual episodes, known as “chills”, with multiple partners sequentially, individually, or in groups. These practices involve the use of various substances, with the intravenous route, known as “slamming” or “slamsex”, being the most dangerous [2,20].

The intravenous route is by far the most dangerous as it facilitates the development of dependence and compulsive use and predisposes to the appearance of local (abscesses, thrombophlebitis) and general (valvulopathies, cellulitis, pulmonary thrombosis) complications. In addition, it enhances the toxic effects of substances and their adulterants in the absence of any prior biological filter. It can also transmit infections (HIV, HCV) in the case of sharing syringes [2].

The lack of information about the risks and consequences of chemsex can result in various adverse outcomes, including intoxication, drug interactions (particularly with antiretrovirals), irritability, insomnia, psychotic symptoms such as delirium or paranoia, accidents, overdose, and an increased risk of sexually transmitted infections (STIs) [1,3,21,22]. Additionally, chemsex has significant social repercussions, often leading to shame, guilt, and ruminative thoughts about excessive spending on parties and drugs. These issues, coupled with a loss of impulse control and behavioral alterations, can turn chemsex into an ineffective coping mechanism, further exacerbating what is already a severe health concern.

Current theoretical contributions on chemsex suggest that multiple interacting factors contribute to the complexity of this phenomenon. However, critical associated factors such as anxiety, depression, risk of psychosis, somatic symptoms, and suicide risk remain underexplored. This research gap may impede health professionals’ ability to identify and address these vital warning signs in chemsex users. To improve their care and overall well-being, it is crucial to identify predictive behaviors and address the specific needs of this population. This underscores the necessity for designing and developing a questionnaire to detect the risk factors associated with chemsex [23]. The Chem-Sex Inventory (CSI) was developed to fulfill this need, providing a tool to assess the psychometric properties of mental health issues among MSM who engage in chemsex, a gap that no existing instrument currently addresses.

This manuscript presents the results of the construct validity analysis phase of the Chem-Sex Inventory (CSI), since the first phase of instrument construction and content validity has been previously published [23].

## 2. Methods

### 2.1. Design

An observational cross-sectional study was carried out between January and April 2023.

### 2.2. Materials

A questionnaire was developed by selecting from a set of items that obtained the best results in content validity when evaluated by a group of experts in chemsex. The initial set of about a hundred items was selected by researchers from different questionnaires used to evaluate emotional instability, psychosis risk, altered body image perception and suicide risk.

Fifty experts on the subject were contacted, defined as professionals with more than five years of experience in their field and at least two years of experience managing users who practice chemsex. These professionals understood the chemsex phenomenon, were active in their field, and had a direct relationship with chemsex users. The group of experts included ten LGBTQ+ individuals, ten mental health professionals, ten emergency care specialists, ten primary care professionals, and ten professionals from infectious disease and STI units. The experts were invited to participate in the study via email, which included an introductory letter to the survey informing about chemsex and its risk factors, as well as an information sheet detailing the study’s characteristics, research objectives, selection criteria, data confidentiality, and the voluntary nature of the study. Of the 50 experts initially contacted, 72% (36 experts) agreed to participate and completed the first round. Only 30 experts (60%) completed the second and final round, of which 70% were men and 30% were women. Notable characteristics of the expert group included eight mental health professionals, five emergency professionals, six primary care professionals, seven infectious disease professionals, and four LGBTQ+ professionals. They came from seven autonomous communities: Aragón (*n* = 1), Extremadura (*n* = 2), Canary Islands (*n* = 2), Catalonia (*n* = 5), Valencian Community (*n* = 1), La Rioja (*n* = 3), and Madrid (*n* = 16). Regarding their profession, 56.66% (*n* = 17) were nurses, 33.33% (*n* = 10) were physicians, 6.66% (*n* = 2) were psychologists, and 3.33% (*n* = 1) were social workers and sexologists.

Following the Delphi method, this expert evaluation was conducted in two rounds [23]. The conventional Delphi method was applied, using an iterative process in which experts are consulted in two rounds [24]. Linstone and Turoff (1975) consider that two rounds are sufficient to reach a consensus, allowing adequate reflection on the group’s responses [25]. This process was developed during September and October 2022 through different phases. In the initial phase of developing a questionnaire on the risk behaviors associated with the chemsex phenomenon, the experts were tasked with evaluating the relevance and comprehensibility of each item using a Likert-type scale from 1 (strongly disagree) to 5 (strongly agree) to refine the aspects and structure of the future questionnaire. Additionally, a qualitative question regarding the relevance and clarity of the sections was included, as well as criteria for completeness, phrasing, and organization of each item. After receiving responses from the experts, a discussion group was convened to consider their suggestions. Consequently, the necessary modifications were incorporated, integrating the experts’ opinions and suggestions, and the final version of the questionnaire was established.

Finally, the experts’ responses were compiled to calculate content validity indices. The content validity of the questionnaire was assessed by calculating the content validity index (CVI) and Aiken’s V for each item. A minimum threshold of 0.6 for CVI and Aiken’s V was established to include items in the questionnaire, providing the criteria for item selection. The indicators were calculated based on the experts’ evaluations. Following the methodology outlined by Polit and Beck [26] and utilized by other authors [27,28,29], three indicators of content validity were calculated for each item (CVI, kappa coefficient (k), and Aiken’s V) based on the ratings provided by the group of experts. The CSI obtained adequate content validity indices, including the items with higher content validity indices, with a global CVI of 0.75 and Aiken’s V of 0.70 [23]. This highlights the global need for tools that comprehensively investigate the critical factors associated with the chemsex phenomenon.

To evaluate the items’ comprehension, experts were asked to assess the degree of understanding for each item during the first round and to recommend any necessary revisions. The average score for each item was then calculated. Items with scores above 4 were classified as highly comprehensible, those between 3.5 and 4 were considered moderately comprehensible, and those below 3.5 were deemed low. Items that received lower scores in the first round but were retained for their content validity were reformulated and reassessed for comprehensibility by the experts in the second round of the Delphi process.

### 2.3. Sample, Settings, and Procedure

The study was conducted in Spain for the calculation, and the recommendations for this type of study were considered, which indicate recruiting between 5 and 10 participants per item with a minimum of 200 participants [30]. Inclusion criteria were (1) over 18 years of age, (2) identify as male, (3) be attracted to men or MSM, and (4) have knowledge of Spanish to complete the questionnaire. Exclusion criteria were (1) unwillingness to sign the informed consent form and (2) participants who filled out the questionnaire incorrectly. All participants completed the complete CSI questionnaire consisting of four dimensions: Dimension 1: emotional instability (15 items); Dimension 2: risk of psychosis (18 items); Dimension 3: altered body perception (5 items); Dimension 4: risk of suicide (4 items). Each item used a five-point Likert scale for responses, with higher scores indicating a higher risk of behaviors associated with the chemsex phenomenon. A sociodemographic questionnaire was also used to collect characteristics and factors related to chemsex practice, such as age, gender identity, sexual identity, marital status, autonomous community, employment status, socio-economic status, and educational level, as well as data related to substance use and PrEP treatment.

The study was conducted in all the autonomous communities of Spain. The questionnaire “The Chem-Sex Inventory (CSI)” was distributed to MSM who consume substances, particularly in a sexual environment. The questionnaire was prepared in a digital version (online questionnaire) using the Microsoft Forms^®^ tool and subsequently migrated to the https://www.estudioenfermeria.com (accessed on 25 January 2023) platform for digital dissemination and IP control to increase methodological control and avoid duplication. Descriptive statistics were computed for all variables (frequency, percentage, mean, standard deviation (SD), and skewness coefficients, where appropriate).

### 2.4. Ethical Considerations

This study was conducted under the Declaration of Helsinki and was approved by the University of La Rioja Committee with verification code (CSV) (D2R1m2Iu3vLVPdIzGZVnK0h6N558tCyN) for human studies through this link: https://sede.unirioja.es/csv/public/index.xhtml;jsessionid=2D8AB94A16FEB14C923EFD5E63C25AF2-n1.ma_07.

Participants were fully informed about the study, and data collection began after signing the informed consent form. The information was treated confidentially and anonymously by dissociating data, following the Data Protection Regulation (EU) 2016/679 of the European Parliament and Organic Law 3/2018. The researchers declare no ethical, moral, or legal conflicts. Participants did not receive any compensation for answering the questionnaire, as it was voluntary.

### 2.5. Data Analysis

Sociodemographic variables were analyzed with descriptive statistics, absolute value, and percentage. CSI factorial structure was analyzed with confirmatory factor analysis (CFA) using a four-factor structure reflecting the four theoretical dimensions (emotional instability, risk of psychosis, altered body perception, and risk of suicide).

To evaluate CFA solutions, based on Hoyle’s [31] recommendations and according to a multifaceted approach to the assessment of the model fit, we used: (a) comparative fit index (CFI [32]), (b) Tucker and Lewis incremental Index (TLI [33]): values greater/equal to 0.90 or better than 0.95 support good fit; (c) root mean square error of approximation (RMSEA [34]): values lower than 0.06 are indicative of a good approximation of fit; and (d) standardized root mean square residual (SRMR [31]): values lower than 0.08 indicate a good fit. Internal consistency of the CSI factors was evaluated using Cronbach’s α [35] and McDonald’s omega [36]. Statistical analysis was conducted using SPSS 21.0 and Mplus 7 software. A *p*-value < 0.05 was considered statistically significant. After performing this analysis, factors with eigenvalues greater than 1 Cronbach’s alpha were considered to evaluate the factors’ internal consistency and the overall consistency of the questionnaire [37]. With the positive results of the AFE, a Confirmatory Factor Analysis (CFA) based on structural equation modeling was performed. To construct the structural equation models, latent variables were created and calculated using the factors obtained in the previous section and the observed variables (items associated with each of the factors obtained).

## 3. Results

**Figure 1:**
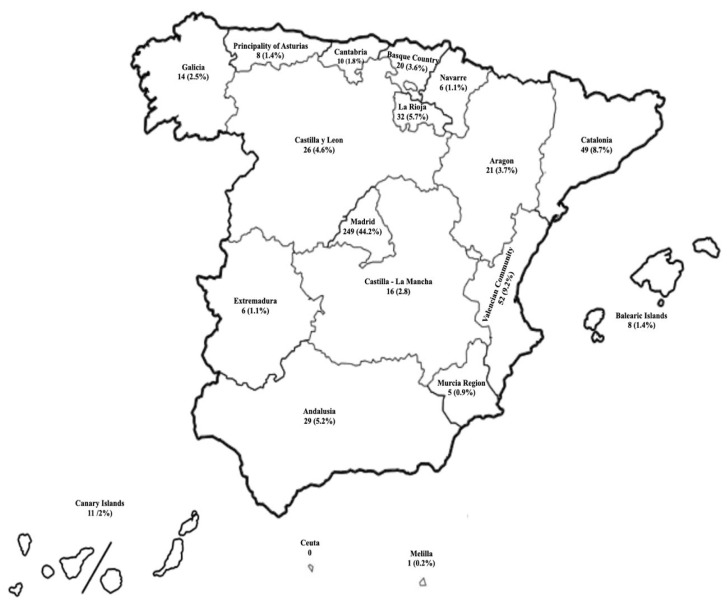
Percentages of chemsex in Spain (*n* = 563).

A total of 563 participants responded to the questionnaire. Of the study participants, 97.7% were cisgender. The mean age was 36 years. The community of Madrid had the highest participation, at 44.4%. Figure 1 shows the percentages of chemsex practice by the autonomous communities in Spain, and Table 1 shows the sociodemographic characteristics of the sample.

### 3.1. Factorial Structure of the CSI

In our study, we conducted a descriptive analysis of all variables to characterize the study population and examine the variables involved. To validate the construct of chemsex behavior, an Exploratory Factor Analysis (EFA) using principal component analysis was performed to identify the dimensions into which the questionnaire items were grouped. These dimensions were identified as follows: Dimension 1—Emotional Instability, Dimension 2—Psychosis, Dimension 3—Altered Body Image Perception, and Dimension 4—Suicide Risks.

To ensure the suitability of the EFA for the study population, Bartlett’s test of sphericity was used and found to be significant (*p* < 0.05), indicating that the correlation matrix was appropriate for factor analysis. Additionally, the Kaiser–Meyer–Olkin (KMO) measure of sampling adequacy was more significant than 0.75, confirming the adequacy of the sample size for the analysis [38]. Specifically, the analysis achieved a KMO of 0.899 and Bartlett’s test of sphericity with a *p*-value of <0.01. The results of the principal component analysis (PCA) of the 42 items, using orthogonal (varimax) rotation, are presented in Table 2.

### 3.2. Factorial Analyses

**Figure 2:**
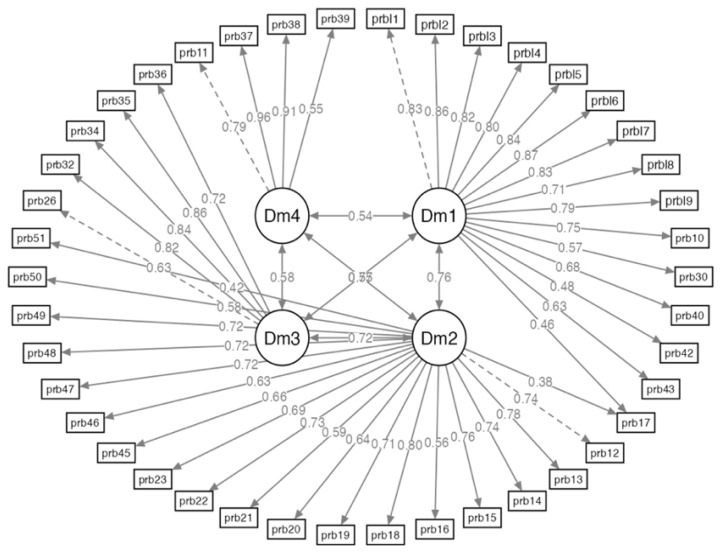
Confirmatory factor analysis of CSI.

We performed an exploratory factor analysis (EFA) based on the four dimensions related to the questionnaire items (see Table 2). The maximum likelihood extraction method was used in combination with a varimax rotation. To decide the best factor solution, we considered the following criteria: (1) factor loadings, (2) the number of items per factor, (3) the interpretability of the solution, (4) the scree plot of the eigenvalues, and (5) the theory underlying the CSI. According to these criteria, the best solution was to identify four dimensions of the CSI. The first dimension included items 1–10, 17, 30, 40, 42, and 43, and was labeled “emotional instability”. The second dimension included items 12–16, 18–23, and 45–51, and was termed “risk of psychosis”. The third dimension, “altered perception of body image”, included items 26, 32, 34–36. Finally, the fourth dimension included items 11, 37–39, and was labeled “suicide risk”. Since the models tested obtained borderline fit indices, we performed a confirmatory factor analysis (CFA) of each of the CSI dimensions (see Figure 2).

A four-factor model was tested based on the theoretical conceptualization of the CSI. The initial model showed an adequate fit as far as all fit indices were as follows: SRMR Scaled 0.072, RMSA 0.069, Confidence Interval (0.069–0.071) *p* < 0.001, Tucker–Lewis Index (TLI) 0.991, Comparative Fit Index (CFI) 0.992. The correlation matrix of the patients’ factors revealed the presence of correlations over 0.80 (see Figure 2).

### 3.3. Reliability and Item Analysis

Internal consistency reliability was computed using Cronbach’s α coefficients and McDonald’s omega. Results presented in Table 3 show an adequate estimator of internal coherence.

**Figure 3:**
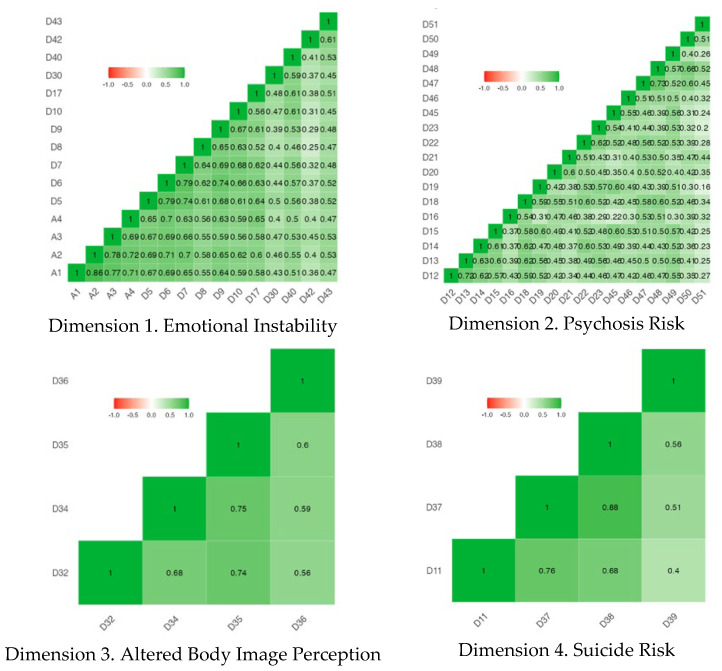
Pearson correlation map.

Pearson’s correlation coefficients showed a significant medium/high correlation in each dimension, determining the validity coefficient. The information functions of each element (dimensions) are shown in Appendix A. All of them provide information, although not to the same extent. See Figure 3.

Appendix A includes the percentile table (10, 25, 50, 75, 90) of the Chem-Sex Inventory (CSI) scale. In addition, this appendix also contains a graphical representation of the dimensions (emotional instability, risk of psychosis, altered body image perception, and suicide risk) that allow us to position the place of the individual visually. Through them, the researcher will obtain an immediate visual representation of the dimensions, discriminating in a simple way, which are the areas in which the individual obtains a lower score. In the same way, he/she will easily discriminate the dimensions in which the person has obtained better scores.

Appendix B includes the final version of the CSI.

## 4. Discussion

This study details the development and validation of a novel tool designed to assist health professionals in assessing the mental health risks associated with chemsex behaviors within the Spanish population. The Chem-Sex Inventory (CSI) questionnaire comprises 42 items, initially developed through an extensive review of global and national research and relevant guidelines. Following this, a content analysis was conducted by a panel of 30 experts, each with at least two years of experience working with chemsex users and over five years of experience in their respective fields, to ensure accurate content validity and comprehension of each item.

This study employed content and construct validation methods alongside reliability testing, including exploratory and confirmatory factor analysis (CFA), to identify structural dimensions and assess internal consistency. The CFA yielded satisfactory reliability values, with Cronbach’s alpha exceeding 0.87 for all dimensions, indicating a good fit across the 42 items.

Notably, this is the first study to develop an instrument specifically aimed at assessing potential behavioral changes related to substance use in chemsex. A comprehensive review informed the development of the CSI of relevant scientific literature and guidelines, including those from the World Health Organization, the Ministry of Health, Social Services and Equality, and the Centers for Disease Control and Prevention, as well as protocols from health centers with specific chemsex procedures. The resulting tool has demonstrated strong psychometric properties, making it a recommended resource for assessing chemsex risk behaviors.

Regarding construct validity, factor analysis is one of the most robust and widely used methods for instrument validation [39]. In this study, the Exploratory Factor Analysis (EFA) with a four-factor model and the Confirmatory Factor Analysis (CFA) revealed a conceptually meaningful pattern of item loadings, indicating that a psychometrically sound questionnaire had been developed. For instance, the association between anxiety and psychosis in the context of chemsex aligns with findings from Dolengevich et al. [40]. While some studies did not find significant differences in anxiety levels among chemsex users, they did report higher scores on the GAD-7 scale [41]. These elevated anxiety levels suggest that substance use to prolong sexual activity may induce depressive symptoms, including feelings of guilt and despair among participants.

Additionally, depressive symptoms such as social isolation, hopelessness, helplessness, or loneliness may serve as triggers for substance use in sexual contexts, where disinhibition provides a means to socialize with others [42]. This highlights the global need for tools that comprehensively investigate the critical factors associated with the chemsex phenomenon.

These gaps in tools to address the chemsex user further confirm not only the existence of universally recognized barriers but also that the Chem-Sex Inventory scale factors have the potential to be applied to different health systems around the world to help assess the mental health risk of chemsex behaviors. The questionnaire proposed in this study is one of the first validated tools that can be used among healthcare professionals with no experience of the chemsex phenomenon. The Chem-Sex Inventory questionnaire contains four significant dimensions to help assess the mental health risks and behaviors of the chemsex user. Adequate knowledge and awareness on the part of professionals is an essential basis for its future implementation. It can help address and intervene in a phenomenon considered a public health problem becoming more prevalent. Knowledge about chemsex in healthcare professionals and adequate awareness can help to detect symptoms early and assess the mental health risk of chemsex behaviors, being an important basis for its future implementation. An extensive literature review helped to generate possible questionnaire items, showing the complex background of the Chem-Sex Inventory (CSI) scale [23].

This questionnaire aimed to analyze a set of chemsex-related risk behaviors using quantitative methods. The items in each dimension explore the connection between each dimension (Dimension 1, Emotional Instability items 1–10, 17, 30, 40, 43; Dimension 2, Psychosis Risk items 12–16, 18–23, 45–51; Dimension 3, Altered body image perception items 32, 34–36; Dimension 4, Suicide Risks items 11, 37–39) and chemsex.

The items of Dimension 1, “emotional instability”, examine the detection of anxiety symptoms and assess the presence of depressive symptoms. Dimension 2, “risk of psychosis”, explores how behavior and conduct may be affected by the practice of chemsex. Dimension 3, “Altered perception of body image”, explores the distortions a person may have about substance use associated with chemsex. Dimension 4, “suicide risk”, examines how chemsex may affect suicidal behavior, impulsivity, and triggers that may lead to completed suicide—showing that it measures consumption behavior, knowing that the higher the score on each of the items, the higher the risk, which characterizes consumption behavior. All authors considered it essential, as it could help quantitatively define the proper disposition to chemsex risk behaviors and could correlate with the other dimensions seen above. The Chem-Sex Inventory (CSI) questionnaire can be used in primary care and hospital centers. Although specific to chemsex, it will be more frequently used in emergency departments, psychiatric services, infectious disease and sexually transmitted infection units, as well as primary care in general. The analysis showed that the developed tool has satisfactory construct and content validity.

Following the above results, we can determine that the Chem-Sex Inventory Scale (CSI) is a valid and consistent tool for assessing risk for chemsex behaviors. The scores obtained on this scale can help health professionals identify chemsex risk behaviors and make targeted interventions. Cronbach’s alpha ranged from 0.87 to 0.95 on all dimensions due to the analysis performed to test the internal consistency of the scale under study. Our research concludes that being a young man living in large cities such as Madrid and Barcelona, where sexual circuits have been established, as well as in gay tourism destinations, being exposed to stressors such as homophobia, rejection, discrimination, and violence that influence people to hide their sexual orientation, and having acquired an STI in the last six months, could be additional risk factors associated with a high prevalence of chemsex. These factors are consistent with other studies showing how the homosexual population is up to twice as likely to suffer psychotic symptoms than the heterosexual population [43,44], chemsex being a coping mechanism for the stressors that MSM experience daily [43,45].

Our study further identifies mephedrone as the most commonly consumed substance intravenously, followed by methamphetamine. This finding aligns with previous research, such as the study by Dolengevich et al., which suggests that methamphetamine is less frequently used due to its higher cost compared to other substances like mephedrone [46]. The consumption patterns are influenced by the substance’s price, purity, form (e.g., powder, pill), and demand.

Focusing specifically on chemsex, prior research has indicated that men aged 36 to 45 years are more likely to engage in chemsex compared to other age groups [47]. Our study corroborates this finding, with the mean age of participants engaging in chemsex being 36 years. A significant finding of our research is the association between chemsex practices and an increased risk of emotional instability, including symptoms of anxiety and depression. These results are consistent with several previous studies among men who have sex with men (MSM), which have reported a link between sexual drug use and a heightened likelihood of depression [7].

Similarly, we found that there are risk factors such as substance use and poly-consumption practice of slamming associated with a greater likelihood of developing a psychotic break. Thus, we also found that there is an association with the risk of psychosis, findings consistent with the studies of Dolengevich et al. and Moreno et al. [40,43]. Other studies that surveyed MSM in the UK and the US reported adverse mental health effects, such as short-term depression and paranoia, following chemsex [42,48]. Other recent review articles by Edmundson et al. and Maxwell et al. identified that most published prevalence studies have not explicitly separated the use of specific substances into use during the chemsex context or outside the sexual context [17,49], so the results of our study allow us to better understand how individuals use specific substances, both in sexual acts and in contexts not associated with the sexual context. A good example of this fact is popper use, which, in our study, we observed doubled in the sexual versus non-sexual context.

The high prevalence of substance use in the context of chemsex, coupled with its association with risky sexual behaviors, underscores the importance of targeting MSM (men who have sex with men) as a critical population for prevention strategies. These strategies should prioritize interventions focused on harm and risk reduction related to chemsex, as well as substance use, more broadly. Specifically, programs should be developed to equip individuals with tools and skills for emotional management and to promote positive acceptance of sexuality, particularly in the face of stigma, rejection, and fear, thereby facilitating the early detection of mental health symptoms.

It is undeniable that chemsex is associated with significant physical, mental, and sexual health problems and may contribute to the spread of sexually transmitted infections (STIs) within the MSM population. Our results indicate a higher prevalence of STIs, particularly gonorrhea and chlamydia, among individuals who engage in chemsex for sexual purposes compared to those who do not. It is essential to clarify that not all sexualized drug use qualifies as chemsex; instead, chemsex refers explicitly to a particular form of sexualized substance use among gay men, bisexuals, MSM, and transgender and non-binary individuals [7].

Related to the above, people living with HIV are at increased risk for mental health problems [42]. An association between chemsex and suicide risk has also been observed, as shown in recent research conducted in the United Kingdom [42,48,50]. A study based on the MSM population has concluded that the lifetime prevalence of suicide attempts in homosexual/bisexual males is two to four times higher than in heterosexual males [42,50]. Along these lines, a recent meta-analysis in Sweden [51] found a 10% rate of suicide attempts in homosexual males versus 2.2% in heterosexual males. These data may suggest that men who have sex with men (MSM) and those who engage in chemsex may have a higher lifetime risk of suicide attempts compared with the general population [41]. In addition, an association has been observed between chemsex and impulsivity, as well as altered body perception. Future studies should explore the interactions between the risk factors identified in chemsex.

The prevalence of chemsex is increasing, particularly in areas with a high population of men who have sex with men (MSM) [52]. Given the addictive nature of chemsex and its associated health risks, it is crucial to prioritize both prevention and treatment efforts that take into account the underlying motivations and psychosocial circumstances driving individuals to engage in this practice. Making chemsex a public health priority is essential.

In countries like the United Kingdom, the National Institute for Health and Care Excellence (NICE) provides health professionals with guidance on psychoactive drugs [52]. The Chem-Sex Inventory scale can play a pivotal role in the early detection of chemsex practices, facilitating effective interventions that address both substance use and the associated harms. Although several attempts have been made to develop questionnaires for health professionals to identify the link between mental disorders and chemsex, no validated scale currently exists to assess the mental health risks associated with chemsex behaviors.

Training healthcare professionals on the nuances of chemsex and substance use can help reduce barriers and stigma, enhancing the visibility of chemsex within health services. This is particularly important given its significant role in mental health issues and HIV/STI risk behaviors. From a public health perspective, the mental health data associated with chemsex are concerning, as mental health problems are among the leading causes of lost productivity and diminished quality of life, with serious health consequences [42,48].

This study has a few limitations: the study design was cross-sectional, with convenience sampling and a relatively homogeneous sample selection. Thus, longitudinal studies would be necessary to evaluate possible changes in behavior and behavior associated with substance use and, specifically, with the chemsex phenomenon. On the other hand, the study was explicitly aimed at substance users, so drug use was high throughout the sample. For this reason, the results should not be used to estimate the prevalence of substance use in the Spanish MSM population. Despite these limitations, this study has the following strengths: it attempted to develop a scale to assess chemsex risk behaviors and their influence on mental health. In addition, the Chem-Sex Inventory scale is a reliable instrument, as the confirmatory factor analysis (CFA) presents adequate reliability values with a Cronbach’s alpha above 0.87 for all dimensions and a good fit of the 42 items. There are no existing scales to evaluate the chemsex phenomenon. In the next research, a cut-off point for the inventory will be established.

### 4.1. Clinical Implications

Since this is the first study that tests the theoretical dimensions of CSI, this study will allow us to understand and comprehend the behaviors and behaviors of chemsex in MSM and to determine the risk of the dimensions evaluated.

### 4.2. Implications for Nursing

Chemsex represents a public health emergency with significant implications for nursing practice across various healthcare settings, including emergency departments, infectious disease services, primary care, and mental health. As a result, nursing professionals must acquire specialized knowledge of chemsex to address this issue effectively. The Chem-Sex Inventory (CSI) is a valuable tool that can assist nurses in identifying and managing the associated risks, thereby enhancing clinical practice in assessment, intervention, and follow-up care. Nurses play a pivotal role in patient education and counseling. With the CSI, they can provide harm reduction strategies and mental health support tailored to the unique needs of chemsex users, ensuring comprehensive and high-quality care.

We are confronted with new patterns of recreational substance use in the sexual context and risk factors that have a clinical impact on the general population, especially in MSM. The possible public health implications of these emerging consumption patterns should be studied, considering the variability in prevalence and the forms of consumption and associated risk factors. Therefore, epidemiological surveillance is necessary to study the impact and evolution of chemsex consumption problems to answer this public health problem and improve prevention and the quality of care for those who practice it.

Training professionals in the approach and management of chemsex can improve the preventive and care response to the health needs of chemsex users. It can also reduce exposure to risk situations regarding infectious diseases and other physical and psychological risks. Concerning the above, studying and identifying the specific risk and protective factors in this particular context of sexualized substance use, as well as interventions, can help to reduce the stigma of this phenomenon, the stigma of mental health and to understand and comprehend the people who practice it.

Some interventions could be focused on providing information about the phenomenon to chemsex practitioners to have a better understanding of their perception of risk concerning the substances used in their practice; other alternatives, as quoted by Raúl Soriano [52], could be to adapt the prevention approach to the local substance market and to incorporate the participation of chemsex users in the discussion on prevention in this area; identifying prevention programs at the national and international level and establishing limits to the advertising of businesses that openly promote chills or chemsex sessions; promoting healthy leisure options that serve as an alternative to counteract the dynamics of hypersexualized leisure already identified in gay men, bisexuals and MSM; as well as analyzing the annual calendar of events of the leisure industry aimed at the gay public as an opportunity for prevention.

Future lines of chemsex research should take advantage of the recent validation of the scale to develop more effective intervention and prevention strategies. Validation of this instrument provides a solid basis for more accurately characterizing individuals at risk for chemsex abuse, thus allowing earlier identification of those who could benefit from preventive measures. This would facilitate the implementation of tailored interventions based on the psychosocial factors identified by the scale, improving the ability to address these risks in a comprehensive manner. In addition, longitudinal studies exploring the prolonged impact of chemsex on mental health, physical health, and the incidence of sexually transmitted infections are of interest.

## 5. Conclusions

Our study has shown that the Chem-Sex Inventory (CSI) scale has factorial validity and could be used in clinical practice and research to measure the behavioral contribution of the chemsex phenomenon in MSM. So far, this has been the first work that has validated a scale to study the chemsex phenomenon.

## Figures and Tables

**Table 1:** Sociodemographic characteristics (*n* = 563).

Variables	*n*	%
Age (mean, SD)	36.9	SD 9.2
Gender identity		
Cisgender	550	97.7
Transgender	2	0.3
Queer (non-binary)	11	2
Sexual Identity		
Gay/Homosexual	372	66.1
Heterosexual	134	23.8
Bisexual	57	10.1
Relationship Status		
Single	276	49
Single with a domestic partner	166	29.5
Married	100	17.8
Divorced	9	1.6
Widower	1	0.2
Others	11	2
Employment status		
Full-time employed	458	81.3
Part-time employed	42	7.5
Unemployed	27	4.8
Retired	4	0.7
Others	32	5.7
Monthly net income		
<499 Euros	14	2.5
500–999 Euros	19	3.4
1000–1499 Euros	99	17.6
1500–1999 Euros	165	29.3
2000–2499 Euros	127	22.6
2500–2999 Euros	67	11.9
3000–4999 Euros	61	10.8
more than 5000 Euros	11	2
Educational level		
Primary school	14	2.5
Baccalaureate	39	6.9
Diploma/Bachelor’s degree	264	46.9
Master’s degree	213	37.8
Doctor of Philosophy (PhD)	33	5.9
Substance use in 12 months in a sexual context		
Amyl nitrite (Poppers)	260	46.2
Medication for erectile dysfunction	111	19.7
GHB/GBL	151	26.8
Alcohol	264	46.9
Tobacco	161	28.6
Ecstasy	67	11.9
Amphetamines	49	8.7
Ketamine	48	8.5
Methamphetamine	58	10.3
Mephedrone	133	23.6
THC	66	11.7
Cocaine	114	20.2
Opioid Analgesics	11	2
Heroin	1	0.2
Substance use in 12 months not in a sexual context		
Amyl nitrite (Poppers)	139	24.7
Medication for erectile dysfunction	7	1.2
GHB/GBL	52	9.2
Alcohol	395	70.2
Tobacco	198	35.2
Ecstasy	75	13.3
Amphetamines	40	7.1
Ketamine	39	6.9
Methamphetamine	27	4.8
Mephedrone	65	11.5
THC	92	16.3
Cocaine	120	21.3
Opioid Analgesics	44	7.8
Heroin	2	0.4
Substances injected in a sexual context in 12 months		
Ketamine	8	1.4
Methamphetamine	21	3.7
Mephedrone	53	9.4
Heroin	1	0.2
PrEP	yes 120	% 21.3
Have you acquired any STIs in the last six months?	yes	%
Gonorrhoea	68	12.2
Chlamydia	53	9.4
Genital herpes	25	4.4
Syphilis	45	8
HIV	17	3
MPOX	10	1.8
Hepatitis	8	1.4

SD: standard deviation; PrEP (pre-exposure prophylaxis); STIs (sexually transmitted infections); HIV (human immunodeficiency virus); MPOX (monkeypox).

**Table 2:** Rotated factor loading of the principal component analysis (PCA) for 42 CSI items (*n* = 563).

**Dimension 1**	**Emotional Instability**																	
**Item**	**1**	**2**	**3**	**4**	**5**	**6**	**7**	**8**	**9**	**10**	**17**	**30**	**40**	**43**	**42**			
**Load**	0.65	0.67	0.65	0.67	0.74	0.79	0.79	0.72	0.78	0.73	0.67	0.46	0.60	0.52	0.46			
**Dimension 2**	**Psychosis Risk**																	
**Item**	**12**	**13**	**14**	**15**	**16**	**18**	**19**	**20**	**21**	**22**	**23**	**45**	**46**	**47**	**48**	**49**	**50**	**51**
**Load**	0.42	0.45	0.47	0.57	0.47	0.63	0.45	0.61	0.66	0.62	0.49	0.49	0.57	0.74	0.73	0.54	0.62	0.51
**Dimension 3**	**Altered body image perception**																	
**Item**	**32**	**34**	**35**	**36**	**26**													
**Load**	0.57	0.55	0.60	0.45	0.42													
**Dimension 4**	**Suicide Risk**																	
**Item**	**39**	**11**	**37**	**38**														
**Load**	0.50	0.63	0.88	0.85														

**Table 3:** Reliability indices of the CSI.

Dimensions	Cronbach’s Alpha	McDonald’s Omega
Dimension 1: Emotional Instability	0.95	0.95
Dimension 2: Psychosis Risk	0.94	0.94
Dimension 3: Altered body image perception	0.88	0.89
Dimension 4: Suicide Risk	0.88	0.89

Dimension 1, Emotional instability items 1–10, 17, 30, 40, 43. Dimension 2, Psychosis Risk items 12–16, 18–23, 45–51. Dimension 3, Altered body image perception items 32, 34–36. Dimension 4, Suicide Risks items 11, 37–39.

## Data Availability

Data are provided upon request to the first author.

## Public Involvement Statement

No public involvement in any aspect of this research.

## Guidelines and Standards Statement

This manuscript used the Consolidated criteria for reporting qualitative research (COREQ) for qualitative research.

## Appendix A. Percentile Chart

**Percentile Chart**				
**n = 563**	**Dimension 1**	**Dimension 2**	**Dimension 3**	**Dimension 4**
Percentile				
10	15.0000	18.0000	5.0000	4.0000
25	20.0000	19.0000	7.0000	4.0000
50	33.0000	24.0000	11.0000	4.0000
75	48.0000	35.0000	16.0000	7.0000
90	57.0000	49.0000	21.0000	13.0000
Dimension 1, Emotional instability. Dimension 2, Psychosis risk. Dimension 3, Altered body imagen perception. Dimension 4, Suicide risk				

## Appendix B. Final Version of the CSI

**Items CSI**
1. Have you felt nervous, anxious or very upset?
2. Have you ever had difficulty relaxing?
3. Have you been easily annoyed or irritated?
4. Have you been afraid as if something terrible was going to happen?
5. Have you had little interest or pleasure in doing things?
6. Have you ever felt discouraged, depressed or hopeless?
7. Have you ever felt tired or low energy?
8. Have you had no appetite or, on the contrary, have you eaten too much?
9. Have you ever felt bad about yourself, felt that you are a failure or that you are failing yourself?
10. Have you had difficulty concentrating on doing everyday things, such as reading the newspaper or watching television?
11. Have you ever had thoughts that you would be better off dead or harming yourself?
12. Do familiar environments sometimes seem strange, confusing, threatening or unreal to you?
13. Have you ever felt as if you were not in control of your own thoughts or ideas?
14. Have ever had difficulty following your own conversation because you ramble or lose concentration too much when you talk?
15. Have you ever had the feeling that other people are watching you or talking about you?
16. Have you occasionally noticed any sensation on or under the skin, such as bugs?
17.Have you ever worried that something might go wrong in your mind?
18. Have you ever felt confused about whether something that happened to you was real or imaginary?
19. Have you ever had feelings of distrust towards other people?
20. Have you ever seen unusual things such as flashes, flames, glaring lights or geometric shapes?
21. Have you ever seen things that other people cannot see?
22. Do you have the feeling that people find it difficult to understand what you are saying?
23. Have you ever had thoughts about your mind going faster than normal?
24. In the last few days have you felt that you were acting impulsively?
25. Do you feel restless in classes or lectures if you have to listen to someone talk for a long period of time
26. Have you ever had chest pains?
27. Have you ever felt that your heart was beating faster than usual?
28. Have you ever felt short of breath?
29. Have you had difficulty sleeping?
30. Have you ever really had the idea of committing suicide?
31. Have you thought about how you would carry it out?.
32. Have you ever tried to take your own life?
33. Have you ever felt that you have difficulty maintaining your attention, that you are easily distracted, or that you are unable to concentrate?
34. Do you consider yourself a person with anger attacks that are difficult to predict?
35. At any time have you considered yourself to be a person who changes moods frequently?
36. Have you ever felt as if people were dropping hints or saying things with a double meaning?
37. Have you ever felt that people look at you strangely because of your appearance?
38. Have you ever felt as if your thoughts were being pulled out of your head?
39. Have you ever felt as if your thoughts were not your own?
40. Have you ever felt as if your thoughts were constantly repeating in your mind?
41. Have you ever felt as if you were under the control of any external force or power?
42. Have you ever heard voices when you were alone?
